# Interlaminar Shear Behavior of Laminated Carbon Fiber Reinforced Plastic from Microscale Strain Distributions Measured by Sampling Moiré Technique

**DOI:** 10.3390/ma11091684

**Published:** 2018-09-11

**Authors:** Qinghua Wang, Shien Ri, Hiroshi Tsuda, Yosuke Takashita, Ryuta Kitamura, Shinji Ogihara

**Affiliations:** 1 Research Institute for Measurement and Analytical Instrumentation, National Metrology Institute of Japan, National Institute of Advanced Industrial Science and Technology, Tsukuba, Ibaraki 305-8568, Japan; ri-shien@aist.go.jp (S.R.); hiroshi-tsuda@aist.go.jp (H.T.); 2 Department of Mechanical Engineering, Tokyo University of Science, Noda, Chiba 278-8510, Japan; yosuketakashita30@gmail.com (Y.T.); rkitamura@rs.tus.ac.jp (R.K.); ogihara@rs.noda.tus.ac.jp (S.O.)

**Keywords:** shear deformation, slippage, delamination, composite materials, strain concentration, Moiré method, phase analysis

## Abstract

In this article, the interlaminar shear behavior of a [±45°]_4s_ laminated carbon fiber reinforced plastic (CFRP) specimen is investigated, by utilizing microscale strain mapping in a wide field of view. A three-point bending device is developed under a laser scanning microscope, and the full-field strain distributions, including normal, shear and principal strains on the cross section of CFRP, in a three-point bending test, are measured using a developed sampling Moiré technique. The microscale shear strain concentrations at interfaces between each two adjacent layers were successfully detected and found to be positive-negative alternately distributed before damage occurrence. The 45° layers slipped to the right relative to the −45° layers, visualized from the revised Moiré phases, and shear strain distributions of the angle-ply CFRP under different loads. The absolute values of the shear strain at interfaces gradually rose with the increase of the bending load, and the sudden decrease of the shear strain peak value implied the occurrence of interlaminar damage. The evolution of the shear strain concentrations is useful in the quantitative evaluation of the potential interlaminar shear failure.

## 1. Introduction

The laminated carbon fiber reinforced plastic (CFRP) [1] have been used in the fields of aerospace, automobiles, and railways, owing to their light weight, high specific strength, and good mechanical properties. However, their applications are still limited, due to their complex damage behaviors [2], such as interfacial debonding, matrix crack and fiber breaking. Their failure behaviors gain more and more attention of researchers and engineers at both meter and micrometer scales. As one of the most common failure modes of the laminated CFRP, delamination is closely related to the interlaminar shear behavior [3], which can be evaluated by the microscale strain distribution. Besides, the conventional structural design applied to metallic structures cannot be used to CFRP structures, due to their quite complex damage behaviors showing complicated strain status. The measurement of microscale strain distribution is important; not only to understand their complex damage behavior, but also to establish a novel structural design for CFRP. Therefore, full-field strain measurement of the laminated CFRP is essential under mechanical loading at the microscale.

Although numerical simulation can be used to analyze the microscale strain distributions of CFRP [4,5,6], experimental measurement is able to provide us with more authentic and credible deformation data. The microscale strain distribution of CFRP cannot be measured from conventionally employed strain gauges owing to the physical size of laminates. Extensometer has been used to measure the nominal strains, including shear strains of different angle-ply CFRP laminates [7], but it can only measure the global strain information instead of the strain distribution. Fortunately, the non-contact and full-field optical methods offer effective ways for measuring microscale strain distributions of CFRP. At present, the optical methods for strain measurement mainly include the Moiré methods, the grid method, the geometric phase analysis (GPA), the digital image correlation (DIC) method, and electronic speckle pattern interferometry (ESPI).

Regarding the studies on strain distributions of CFRP by the optical methods, the strain distributions measured by DIC have been reported for investigating the nonlinear shear deformation of [±45°]_2s_ CFRP [8] and the shear performance of [±45°]_4s_ CFRP [9]. These strain distributions refer to the deformation on the front surface of the laminate, rather than the deformation on the cross section of the laminate, which is also important for instability evaluation. The normal and shear strain distributions across the thickness on the cross sections of [0°/±45°/90°] CFRP laminates [10] have been investigated by DIC, and shear strain localizations have been observed at several interfaces in an uniaxial tension test. Nevertheless, DIC measurement is easily affected by noise as the deformation carrier is speckle. Shear strain measurement of laminated CFRP by ESPI [11] has also been reported; however, ESPI is very sensitive to vibration and difficult to measure large deformation. To the authors’ knowledge, there are few reports on the interlaminar shear strain measurement of [±45°] CFRP by the Moiré methods, the grid method and GPA, since a periodic pattern is necessary to be fabricated. However, with the development of grid fabrication techniques, it becomes easy to fabricate a grid on CFRP. The Moiré methods [12,13] have been widely used in the deformation measurement of composite materials [14], including CFRP [15,16,17,18]. Due to the advantages of deformation visualization, high noise resistance and large field of view, the Moiré methods are at the center of attention, and expected to be used to visualize the shear strain concentration of CFRP.

The Moiré methods can also be subdivided into the geometric Moiré method [12,19], Moiré interferometry [12,18,20], the digital Moiré [17,21] or overlapping Moiré method, the laser scanning Moiré method (also called as laser scanning confocal microscopy Moiré) [22,23], the electron scanning Moiré method (also called as electron beam Moiré or scanning electron microscope Moiré) [24,25], the atomic force microscope Moiré method [26], the sampling Moiré method [27,28], etc. Among the (previously mentioned) various Moiré methods, the sampling Moiré method adopts the spatial phase-shifting technique, and the other Moiré methods are usually combined with the temporal phase-shifting technique to analyze the Moiré phase for deformation measurement. Since the spatial phase-shifting technique does not need any expensive temporal phase-shifting device, such as a piezoelectric motor and is applicable to a single grid image, the sampling Moiré method has been chosen to measure the strain distributions of CFRP.

The sampling Moiré method is usually used for displacement measurement of large infrastructures, such as bridges and buildings. For micro/nano-scale strain distribution measurement, the sampling Moiré method has got some development and several sub-family methods have been successively proposed in recent years. The reconstructed multiplication Moiré method from 2-pixel sampling Moiré allows high-sensitivity deformation measurement in a large field of view [29]. The combination between the sampling Moiré method and the phase differential way capacitates accurate normal strain measurement and has been used for deformation measurement of CFRP [16]. The two-dimensional (2D) Moiré phase analysis method enables both high-accuracy shear strain and normal strain measurement and has been applied for crack occurrence prediction [30]. The Fourier transform filtered sampling Moiré method has strong anti-noise ability and has been used for defect detection [31] and strain evaluation [32] of atomic structures. Recently, a local phase unwrapping algorithm [33] has been proposed to be combined with the 2D Moiré phase analysis method to minimize the influence of defects on strain measurement. When the specimen grid includes two perpendicular gratings and one grating is parallel to the analysis direction, the 2D Moiré phase analysis method can be simplified.

In this work, the developed sampling Moiré technique by combining the local phase unwrapping algorithm and the sampling Moiré method is first used to measure the microscale strain distributions of a [±45°]_4s_ laminated CFRP specimen. To ensure in-situ strain measurement of CFRP in a three-point bending test, a mechanical loading device is developed under a laser scanning microscope. The evolution of the measured shear strain distributions is used to evaluate the interlaminar shear behavior and compared with the damage characteristic of the angle-ply CFRP under three-point bending.

## 2. Principle of Strain Measurement

### 2.1. Phase Extraction from Sampling Moiré Method

**Figure 1:**
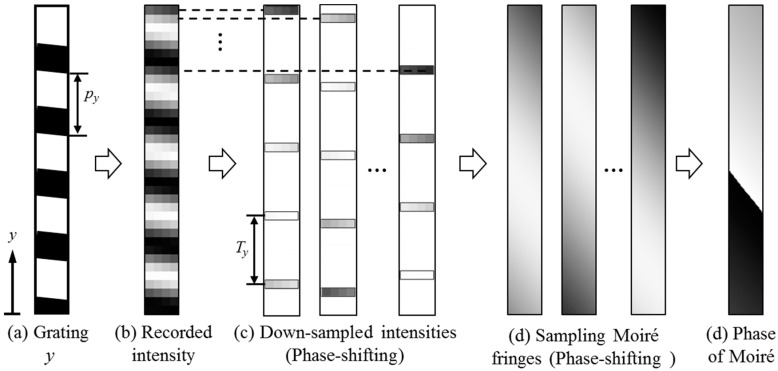
Phase measurement principle of the sampling Moiré method.

A grid can be treated as a combination of two parallel gratings. To simply the expressions of Moiré phase extraction from the 2D Moiré phase analysis method, the Moiré phase extraction method in one direction from a parallel grating is introduced in this section (Figure 1). Suppose the pitch of a grating in the *y* (vertical upwards) direction is *p_y_*, the grating intensity can be expressed as

(1) $$I_{y}=A\cos(2\pi \frac{y}{p_{y}})+B=A\cos\varphi +B$$

where *A* is the modulated amplitude of the grating, *B* means the background intensity and *φ* stands for the phase of this grating.

Spatial phase-shifting Moiré fringes in the *y* direction can be generated from *T_y_*-pixels down-sampling and linear or 2nd-order or 3rd-order intensity interpolation, where *T_y_* expresses the sampling pitch close to the grating pitch. The intensity of phase-shifting Moiré fringes can be represented by

(2) $$\begin{array}{ll} I_{my}(k_{y}) & =A\cos[2\pi (\frac{y}{p_{y}}-\frac{y}{T_{y}}+\frac{k_{y}}{T_{y}})]+B\\ & =A\cos[\varphi_{my}+2\pi \frac{k_{y}}{T_{y}})]+B\;\left(k_{y}=0,\;1,\ldots ,\;T_{y}-1\right) \end{array}$$

where *φ_my_* is the phase of the Moiré pattern when *k_y_* = 0 in the *y* direction, and *k*_y_ means the starting point of the down sampling [27].

The Moiré phase *φ_my_* in the *y* direction can be calculated from the phase-shifting method using a discrete Fourier transform (DFT) algorithm using the following expression [27]:

(3) $$\varphi_{my}=-\arctan\frac{\sum_{k_{y}=0}^{T_{y}-1}I_{my}(k_{y})\sin(2\pi k_{y}/T_{y})}{\sum_{k_{y}=0}^{T_{y}-1}I_{my}(k_{y})\cos(2\pi k_{y}/T_{y})}$$

Similarly, from a grating in the *x* direction, the Moiré phase *φ_mx_* in the *x* direction can also be obtained using the above equations by changing the direction symbol *y* to *x*.

Since the Moiré phase difference is theoretically equal to the grating phase difference in either direction, both the Moiré phase difference or the grating phase difference can be used for deformation measurement. The phase differences before and after deformation are determinable by

(4) $$\begin{array}{l} \Delta \varphi_{x}=\Delta \varphi_{mx}=\mathrm{wrapped}(\varphi {’}_{mx}-\varphi_{mx})\\ \Delta \varphi_{y}=\Delta \varphi_{my}=\mathrm{wrapped}(\varphi {’}_{my}-\varphi_{my}) \end{array}$$

where *φ*’*_mx_* and *φ*’*_my_* stand for the Moiré phases after deformation in the *x* and *y* directions, respectively.

### 2.2. Strain Measurement Using Local Phase Unwrapping Algorithm

**Figure 2:**
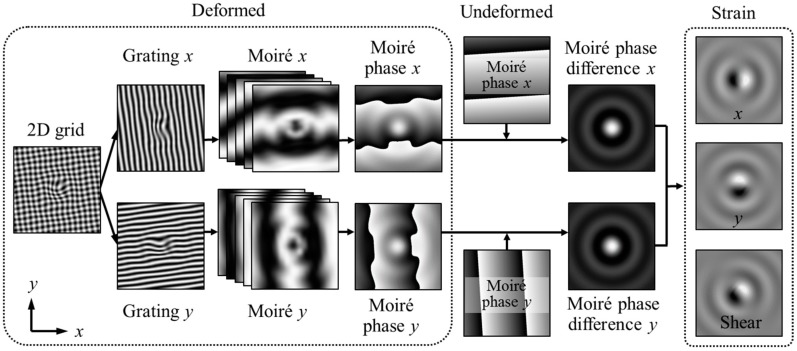
Two-dimensional (2D) strain measurement process, from a 2D grid.

The 2D strain measurement process from a 2D grid is illustrated in Figure 2. The 2D grid can be separated to grating *x* and grating *y* using a low pass filter or Fourier transform. Then, the spatial phase-shifted sampling Moiré fringes can be generated and the Moiré phase differences before and after deformation in the *x* and *y* directions can be calculated as described in Section 2.1. Next, the 2D strain is measurable from the partial differentials of the Moiré phase differences in the *x* and *y* directions. As the phase differences in Equation (4) are wrapped by 2π in the range of (−π, π), the partial differentials of Moiré phase differences in different directions around phase boundaries should be corrected by a local phase unwrapping algorithm [33], expressed as

(5) $$\frac{\partial \Delta \varphi_{i}}{\partial x}=\left\{ \begin{array}{l} \frac{\Delta \varphi_{i}\left(x+1,y\right)-\Delta \varphi_{i}\left(x-1,y\right)}{2}-\pi \;\;(\mathrm{if}\;\frac{\partial \Delta \varphi_{i}}{\partial x}>c)\\ \frac{\Delta \varphi_{i}\left(x+1,y\right)-\Delta \varphi_{i}\left(x-1,y\right)}{2}+\pi \;\;(\mathrm{if}\;\frac{\partial \Delta \varphi_{i}}{\partial x}<-c) \end{array}\right.$$

(6) $$\begin{array}{ll} \frac{\partial \Delta \varphi_{i}}{\partial y}= & \left\{ \begin{array}{l} \frac{\Delta \varphi_{i}\left(x,y+1\right)-\Delta \varphi_{i}\left(x,y-1\right)}{2}-\pi \;\;(\mathrm{if}\;\frac{\partial \Delta \varphi_{i}}{\partial y}>c)\\ \frac{\Delta \varphi_{i}\left(x,y+1\right)-\Delta \varphi_{i}\left(x,y-1\right)}{2}+\pi \;\;(\mathrm{if}\;\frac{\partial \Delta \varphi_{i}}{\partial y}<-c) \end{array}\right.\\ & \left(i=x,\;y\right) \end{array}$$

where *c* represents the critical value of the partial differentials and can be chosen within (0.7π, π).

From the corrected partial differentials of Moiré phase differences, the normal and shear strains can be calculated using the following equation:

(7) $$\begin{array}{l} \varepsilon_{xx}=-\frac{1}{2\pi }\frac{\partial \Delta \varphi_{x}}{\partial x}p_{x}\\ \varepsilon_{yy}=-\frac{1}{2\pi }\frac{\partial \Delta \varphi_{y}}{\partial y}p_{y}\\ \gamma_{xy}=-\frac{1}{2\pi }(\frac{\partial \Delta \varphi_{x}}{\partial y}p_{x}+\frac{\partial \Delta \varphi_{y}}{\partial x}p_{y}) \end{array}$$

The above equation can be regarded as a simplified expression of the strain measurement equation in [30] or [33] by considering that the 2D grid includes two perpendicular gratings and one grating is parallel to the analysis direction.

After the normal and shear strains are obtained, for the plane stress problems, the maximum and minimum principal strains are measurable by

(8) $$\begin{array}{l} \varepsilon_{\max}=\frac{\varepsilon_{xx}+\varepsilon_{yy}}{2}+\sqrt{{\left(\frac{\varepsilon_{xx}-\varepsilon_{yy}}{2}\right)}^{2}+\frac{\gamma_{xy}^{2}}{4}}\\ \varepsilon_{\min}=\frac{\varepsilon_{xx}+\varepsilon_{yy}}{2}-\sqrt{{\left(\frac{\varepsilon_{xx}-\varepsilon_{yy}}{2}\right)}^{2}+\frac{\gamma_{xy}^{2}}{4}} \end{array}$$

Consequently, all the full-field normal, shear and principal strains can be calculated using the developed sampling Moiré method.

## 3. Materials and Experiments

### 3.1. Specimen Preparation and Grid Fabrication

**Figure 3:**
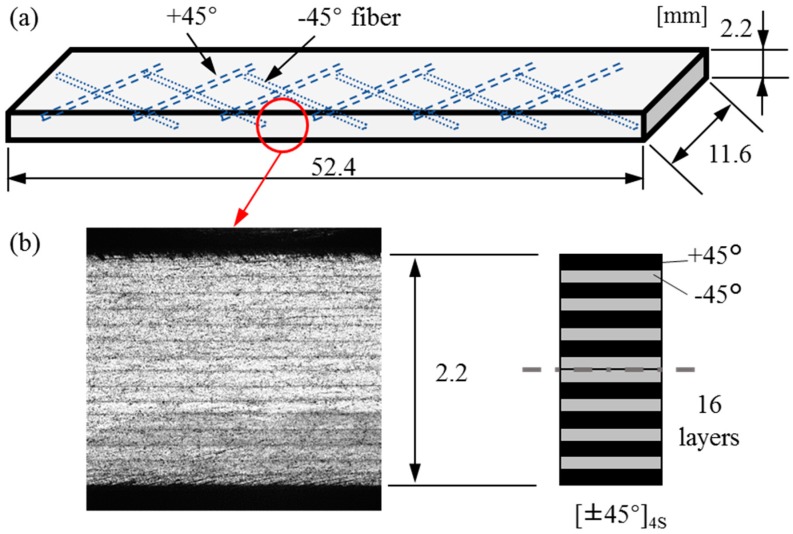
Carbon fiber reinforced plastic (CFRP) specimen geometry: (**a**) Specimen size and fiber directions; (**b**) surface image and layer structure on cross section.

The specimen was [±45°]_4S_ laminated CFRP with the width of 11.6 mm, length of 52.4 mm and thickness of 2.2 mm (Figure 3a). The materials were T700SC/2500 carbon/epoxy (Toray Co., Ltd., Tokyo, Japan) prepregs [34]. There were 16 layers (16-ply prepregs) and the thickness of each layer was 0.14 mm. First, 8 layers were alternately stacked with the stacking sequence of +45° and −45°. Then, another 8 layers were symmetrically stacked with the stacking sequence of −45° and +45° from the 9th layer to the bottom layer (Figure 3b), to form a balanced structure with less coupling effect [35]. The prepregs were cured in an autoclave at a preheating temperature of 90 °C for 1h followed by heating at 130 °C at a pressure of 0.2 MPa for 3.5 h, and the cured laminate was cut to the specimen with the desired size using a composite material cutting machine (AC-300CF, Maruto Testing Machine Co., Tokyo, Japan) [35].

**Figure 4:**
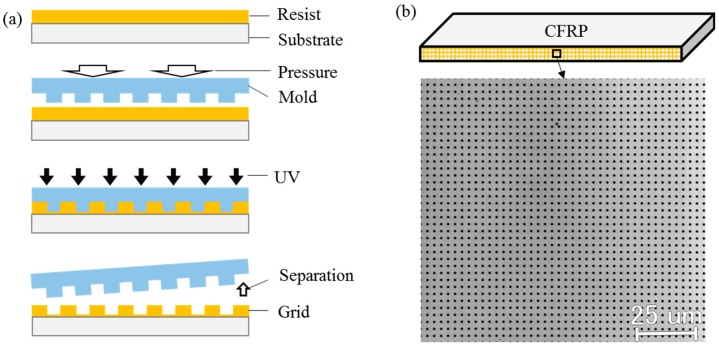
Microscale grid fabrication: (**a**) Process of ultraviolet (UV) nanoimprint lithography; (**b**) grid image fabricated on CFRP specimen.

The 52.4 × 2.2 mm^2^ surface was polished by sandpaper with size of #800 and polishing solutions (DP-Spray P 15 μm followed by P 1 μm). On the polished surface, a 3-μm-pitch grid was fabricated in an ultraviolet (UV) nanoimprint lithography device (EUN-4200, Engineering System Co., Ltd., Matsumoto, Japan). The grid fabrication process of UV nanoimprint lithography is illustrated in Figure 4a. The used nanoimprint mold was a commercial grid mold from SCIVAX Corporation (Kawasaki, Japan). The used resist was PAK01, the UV wavelength was 375 nm and the exposure time was 30 s. The fabricated grid image is shown in Figure 4b observed with a laser scanning microscope (Lasertec Hybrid Optelics, Yokohama, Japan). The grid contains two orthogonal gratings, which are respectively parallel and perpendicular to the specimen axial direction.

### 3.2. Three-Point Bending Test

**Figure 5:**
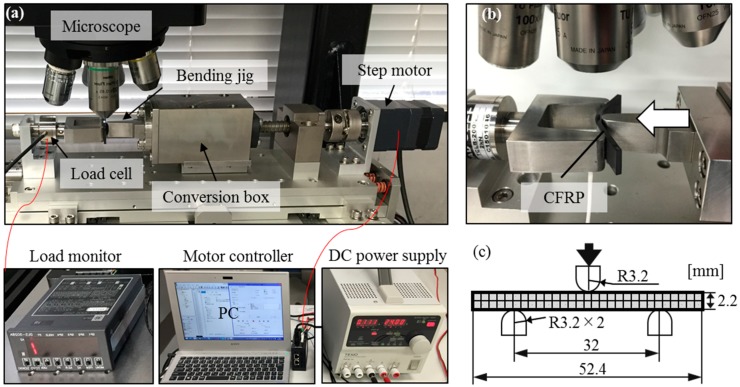
Experimental setup: (**a**) Developed three-point bending device under a laser microscope; (**b**) enlarge image of the loading unit; (**c**) loading size.

To perform a three-point bending test to the laminated CFRP, an automatic mechanical loading device was designed under the laser scanning microscope. The designed device includes a stepper motor, a motor controller, a rotation-translation conversion box, a pair of bending jig, a load cell, a load monitor and a direct current (DC) power supply, as shown in Figure 5. The model of the stepper motor was CRK543AKD-H100 (Oriental Motor Co., Ltd., Tokyo, Japan) and the designed minimum translational incremental step of the loading jig was 80 nm. The loading speed of the movable loading head during the three-point bending test was set to be 10 μm/s.

The radiuses of the movable and fixed loading heads were 3.2 mm, and the support span was set to 32 mm, near 16 times of the specimen thickness in accordance with the American Society for Testing and Materials (ASTM) standards (Figure 5c). During the three-point bending test, the bending load was measured by the load cell (TCLB-200L, Toyo Sokki Co., Ltd., Yokohama, Japan) with the maximum measurable load of 2 kN, and the grid images were recorded in situ by the laser microscope.

## 4. Results and Discussion

### 4.1. Normal, Shear and Principal Strain Distributions

**Figure 6:**
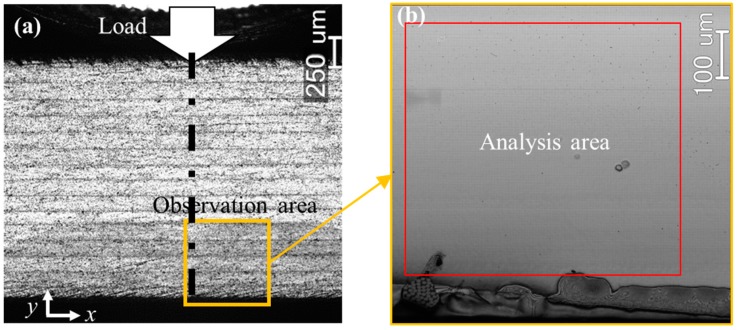
Surface images showing (**a**) the observation area and (**b**) the analysis area on CFRP specimen during three-point bending test.

Prior to the experiment of the [±45°]_4s_ angle-ply CFRP specimen investigated in this study, a three-point bending test was performed to another [±45°]_4s_ CFRP, which was manufactured using the same curing and cutting processes, but no grid was fabricated on. During the bending test, the surface of CFRP without grid was observed using the laser microscope at different magnifications, and it was found that the most vulnerable part (crack and delamination locations) was not directly below the loading head, but slightly deviated from the extension line of the load. Therefore, in this study, an area near the bottom surface of the CFRP specimen, which slightly deviates from the load extension line was selected as the observation area (Figure 6a). A region with the size of 600 × 550 µm^2^ in the observed grid image was chosen as the region of interest (ROI), as seen in Figure 6b. The axial direction of CFRP was defined as the *x* direction, as shown in Figure 6a. The normal, shear and principal strain distributions under different bending loads were measured using the developed sampling Moiré method.

**Figure 7:**
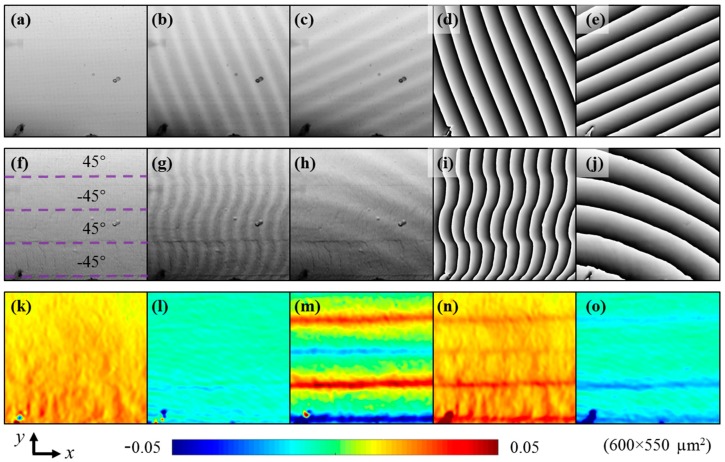
Strain measurement process and results: Images of (**a**) grid, (**b**) Moiré *x*, (**c**) Moiré *y*, (**d**) Moiré phase *x* and (**e**) Moiré phase *y* at 0MPa; Images of (**f**) grid, (**g**) Moiré *x*, (**h**) Moiré *y*, (**i**) Moiré phase *x* and (**j**) Moiré phase *y* at 258 MPa; distributions of (**k**) strain *x*, (**l**) strain *y*, (**m**) shear strain, (**n**) maximum principal strain and (**o**) minimum principal strain of CFRP. The phase range is −π–π, and the symbols *x* and *y* are abbreviations of ‘in the *x* direction’ and in the *y* direction’, respectively.

Figure 7 illustrates the strain measurement process and results with an example when the three-point bending load is 316 N, i.e., the maximum bending stress at the bottom surface is 258 MPa. As the grid pitch (3 μm) was around 4.2 pixels in the recorded image before deformation, the sampling pitch *T* = 4 pixels (*Tx* = *Ty*) was used to generate Moiré fringes using third order intensity interpolation. The Moiré fringes, as well as the corresponding Moiré phases in the *x* and *y* directions were respectively obtained from the grid images at 0 MPa and 258 MPa. A Sin/Cos filter [36] with a half kernel size of 3 pixels was applied to smooth these Moiré phases. The tortuous Moiré fringes and Moiré phase in the *x* direction, after deformation, in Figure 7g,i indicate the non-uniform deformations of different layers, where the greater phase change or deformation can be observed in the lower layer interface. Then, the Moiré phase differences in the *x* and *y* directions were obtained and the 2D strain distributions, including normal, shear and principal strains were calculated using Equations (7) and (8). An average filter with the size of 13 × 13 pixels was used to smooth all the strain distributions. 

As seen from Figure 7, the normal strain in the *x* direction in Figure 7k is tensile and several spindly strain concentrations occur in the bottom layer along with the vertical direction, i.e., loading direction. The normal strain in the *y* direction in Figure 7l is compressive and weak strain concentrations can be seen along with the layer interface. The shear strain in Figure 7m is most interesting, positive and negative strain concentrations appear alternately at layer interfaces. It indicates that the slip deformation occurs at different layers. The maximum and minimum principal strain concentrations in Figure 7n,o reflect the comprehensive features of normal and shear strain concentrations. The maximum principal strain (Figure 7n) concentrates at the horizontal layer interfaces where the shear strain concentration occurs (Figure 7m), and, furthermore, concentrates at some spindly vertical regions where the concentration of the strain in the *x* direction emerges (Figure 7k). It indicates that damage is likely to occur at the layer interfaces and these vertical spindly regions, since the maximum principal strain is tensile.

The normal, shear and principal strain distributions at other loads were also measured from Moiré phases, which show similar distribution features as in Figure 7k–o. The evolution of the Moiré phase enables us to observe the interlaminar slip deformation intuitively, and the evolution of the shear strain can be used to evaluate the interlaminar shear behavior of the specimen and predict the delamination occurrence location, which will be elucidated in Section 4.2 and Section 4.3, respectively.

### 4.2. Visualization of Relative Slip Direction of Different Layers

Since Moiré fringe is an amplification phenomenon of the specimen grid and the Moiré phase has the same distribution as the Moiré fringe, such as Figure 7g,i, the Moiré phase can also be considered as an amplification pattern of the specimen grid. Therefore, the tiny slip deformation of the specimen can be visualized from the Moiré phase. However, it is noteworthy that the distribution trend of the Moiré phase is identical to that of the grid when the sampling pitch is greater than the grid pitch, whereas the distribution trend of the Moiré phase is opposite when the sampling pitch is smaller than the grid pitch [31]. As the used sampling pitch is *T* = 4 pixels, smaller than the grid pitch on CFRP (around 4.2 pixels), the Moiré phases in Figure 7 should be revised, by using a sampling pitch greater than the grid pitch to visualize the slip deformation of the laminated CFRP.

**Figure 8:**
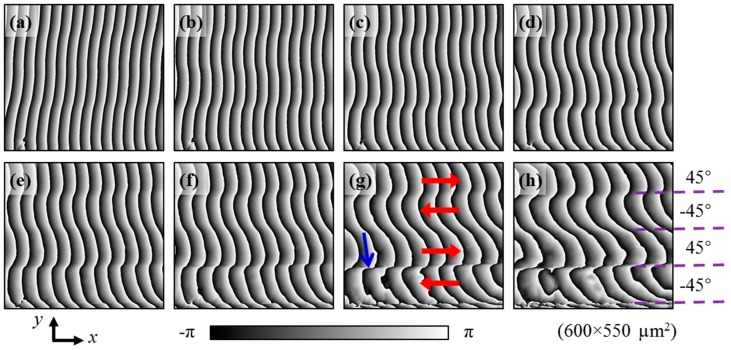
Visualization of the relative slip directions of different layers from revised Moiré phases in the *x* direction at (**a**) 127 MPa, (**b**) 199 MPa, (**c**) 238 MPa, (**d**) 258 MPa, (**e**) 272 MPa, (**f**) 292 MPa, (**g**) 313 MPa, and (**h**) 327 MPa.

Because slippage occurs along the CFRP layer length direction (*x* direction), only the Moiré phase in the *x* direction is revised to visualize the slip deformation. The revised Moiré phase in the *x* direction can be obtained by $\varphi_{mx\_\mathrm{rev}}=\varphi_{x}-2\pi x/T_{\mathrm{rev}}=(\varphi_{mx}+2\pi x/T)-2\pi x/T_{\mathrm{rev}}$, where *φ_x_* is the grid phase and *φ_mx_* is the calculated Moiré phase in the *x* direction. A revised sampling pitch *T*_rev_ = 4.5 pixels is adopted to get the revised Moiré phases in the *x* direction, as shown in Figure 8, for visualization of the slip deformation under different loads, i.e., the maximum bending stresses at the bottom surface are 127 MPa, 199 MPa, 238 MPa, 258 MPa, 272 MPa, 292 MPa, 313 MPa, and 327 MPa, respectively.

It is clearly seen that, the 45° layer slips to the right and the −45° layer ships to the left relatively. With the increase of the three-point bending load, the slip deformation becomes severe. The slip deformation at the (45°/−45°) layer interfaces is more severe than that at the adjacent middle (−45°/45°) layer interface. The most violent slip deformation appears at the bottom (45°/−45°) layer interface pointed out by the blue arrow in Figure 8g. All the slip deformation can be quantitatively evaluated by the shear strain distribution illustrated in Section 4.3. As the Moiré phase difference is equal to the difference of the revised Moiré phases before and after deformation, the strain can be calculated by either the Moiré phase difference or the revised Moiré phase difference.

### 4.3. Evolution of Shear Strain Distribution

**Figure 9:**
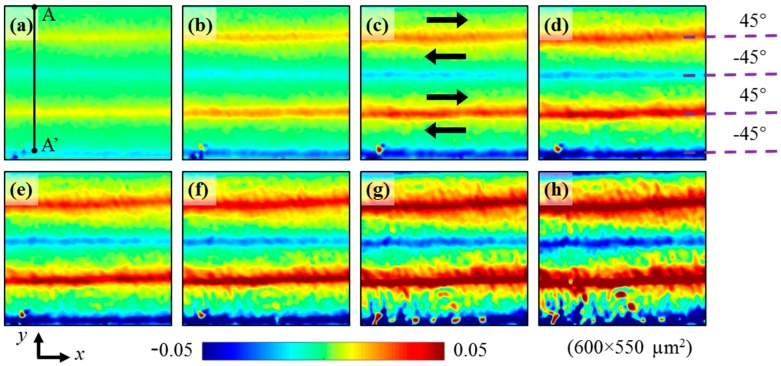
Evolution of the shear strain distributions of CFRP at (**a**) 127 MPa, (**b**) 199 MPa, (**c**) 238 MPa, (**d**) 258 MPa, (**e**) 272 MPa, (**f**) 292 MPa, (**g**) 313 MPa, and (**h**) 327 MPa, where the arrows show the relative slip directions of different layers.

Using the same measurement procedure and parameters as in Section 4.1, the shear strain distributions of CFRP at different three-point bending loads were calculated. The evolution of the shear strain distribution is presented in Figure 9, when the maximum bending stresses at the bottom surface are 127 MPa, 199 MPa, 238 MPa, 258 MPa, 272 MPa, 292 MPa, 313 MPa, and 327 MPa. The shear strain concentrations at layer interfaces at different loads reveal the large shear deformation between each two adjacent layers.

The shear strain is positive at (45°/−45°) layer interface, indicating the upper 45° layer slips to the right relative to the lower −45° layer. The shear strain is negative at (−45°/45°) layer interface, indicating the upper −45° layer slips to the left relative to the lower 45° layer. The relative slip direction determined from the shear strain in Figure 9 is the same as the slip direction visualized from the revised Moiré phases in Figure 8. The shear strain distribution enables quantitative evaluation of the interlaminar shear behavior of CFRP.

When the three-point bending stress is 127 MPa, the absolute values of the shear strain at (45°/−45°) and (−45°/45°) layer interfaces are almost the same, showing the symmetry of slippage. However, when the bending stress increases, the positive shear strain at (45°/−45°) layer interface grows faster than the absolute value of the negative shear strain at (−45°/45°) layer interface, attributed to the influence of the tensile strain in the *x* direction. The shear strain is maximum at the lower (45°/−45°) layer interface.

**Figure 10:**
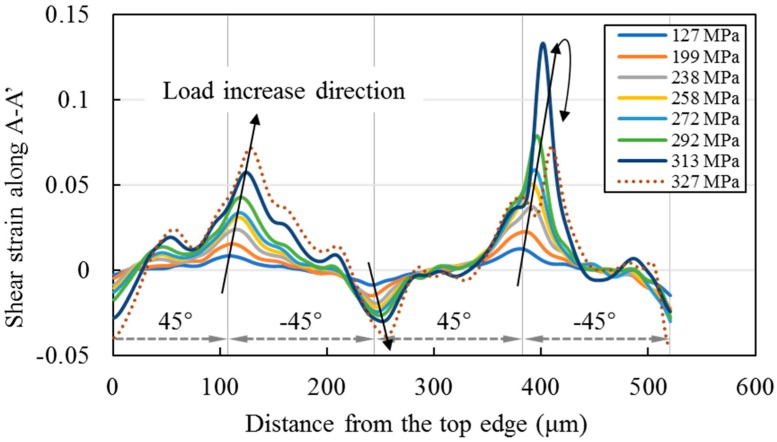
Shear strain of CFRP under different loads along the section line A-A’ labeled in Figure 9a.

To display the shear strain feature more clearly, the strain data along a section line A-A’ labeled in Figure 9a is plotted with the increase of the three-point bending stress, as illustrated in Figure 10. To avoid showing the strain data, which may be influenced by the defect at the bottom-left corner of the grid image, the length of A-A’ is set to be 520 μm, shorter than the total height 550 μm. It can be seen from Figure 10, at the upper two layer interfaces, the absolute values of the shear strain gradually increase with the increase of the bending stress. At the lower (45°/−45°) layer interface, the shear strain also gradually increases with the load increase when the bending stress is less than 313 MPa, and the peak value is greater than those at the upper two interfaces. However, when the bending stress increases from 313 MPa to 327 MPa, the shear strain decreases, and the peak value decreases from 0.133 to 0.072 for this tested specimen.

**Figure 11:**
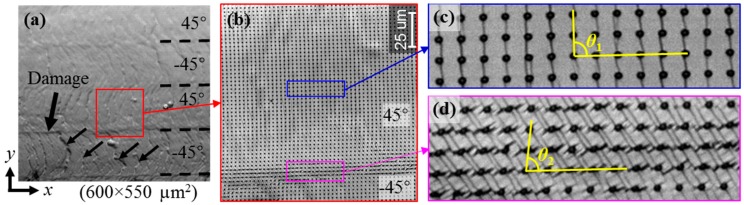
Recorded 2D grid images in (**a**) the analysis area and (**b**) a local area near the lower (45°/−45°) layer interface, and enlarged views (**c**) in the middle of 45° layer and (**d**) at the (45°/−45°) layer interface on CFRP at 327 MPa in the three-point bending test.

The sudden decrease of the shear strain peak value at the lower (45°/−45°) layer interface is attributable to a damage occurrence, which causes release of a part of shear stress. The emerged damage at the lower (45°/−45°) layer interface can be verified from the grayscale change observed from the surface image of CFRP under 327 MPa labeled by an arrow in Figure 11a. It can be deduced from Figure 9, Figure 10 and Figure 11, interlaminar damage arises when the shear strain is greater than 0.133 for the single specimen of this study. This damage is likely to be the source of future delamination. It demonstrates that the shear strain concentration is useful in prediction of the interlaminar delamination location. However, for investigating the delamination initiation strains in the material system, the strain distributions of more than three specimens should be measured.

Besides the interlaminar damage, lots of numerous micro damages along the vertical direction emerge at the bottom −45° layer, shown in Figure 11. That is because of the tensile strain concentration regions of the strain in the *x* direction and the maximum principal strain, as seen in Figure 7k,n. The tensile strain concentration is likely to cause damage such as crack within layers.

Furthermore, the relative slip direction of different layers analyzed in Section 4.2 can be verified from the enlarged view of the microgrid observed by using a high magnification objective lens. The grid dots before deformation are distributed along horizontal and vertical lines, as seen in Figure 4. The grid dots after deformation are distributed along oblique lines, which can be observed from the enlarged 2D grid images on CFRP at 327 MPa shown in Figure 11b–d. Since the ROI is at the bottom right area below the bending head, the grid should rotate anticlockwise slightly when there is less shear deformation, as seen from the grid image in the middle area of the 45° layer (Figure 11c). However, the grid along the vertical line rotate clockwise at the (45°/−45°) layer interface (Figure 11d). It indicates that shear deformation occurs at the (45°/−45°) layer interface and the 45° layer slips to the right relative to the −45° layer, which is identical to the direction visualized in Figure 8.

The magnitude order of the measured shear strain in Section 4.3 can also be verified by the estimation of the shear strain from the enlarged grid image in Figure 11. The grid angle is 90° before deformation from Figure 4. The shear strain can be estimated from the angle reduction of the microgrid due to deformation, since the tangent of a small angle is approximately equal to this small angle. In the middle area of the 45° layer, the grid angle *θ*_1_ is still close to 90° at 327 MPa (Figure 11c) and the angle change is near zero, suggesting that the shear strain is near zero in accord with the measured shear strain in Figure 9h. At the (45°/−45°) layer interface, the grid angle *θ*_2_ is close to 82.5° at 327 MPa (Figure 11d) and the angle reduction is near 7.5°, i.e., 0.13 in rad. It means that the shear strain at (45°/−45°) layer interface is around 0.13, the magnitude order of, which is the same to that of the measured shear strain in Figure 9h and Figure 10.

### 4.4. Discussion

The phenomenon of the shear strain concentrations at the layer interfaces on the cross section of [±45°]_4s_ CFRP found in this study agrees well with the results in other experimental [10] and numerical studies [37]. The shear strain distributions across the thickness of [0/−45°/45°/90°] and [0/90°/45°/−45°] laminates in an uniaxial tension test were measured by DIC in [10], and the highest interlaminar shear strain occurred at the interfaces between −45° and 45° layers. The distribution trend of the shear strain of [±45°]_4s_ laminate under three-point bending in this study is similar to that of the [0/−45°/45°/90°] laminate under tensile bending in [10], i.e., the shear strain is positive at the (45°/−45°) layer interface and negative at the (−45°/45°) layer interface (Figure 9 and Figure 10). Whereas this distribution trend is opposite to that of the [0/90°/45°/−45°] laminate, i.e., the shear strain of the [0/90°/45°/−45°] laminate under tensile bending in [10] is negative at the (45°/−45°) layer interface and positive at the (−45°/45°) layer interface. Whatever, it is known that the shear strain tends to concentrate at the (45°/−45°) and (−45°/45°) layer interfaces, and the combined effect of the interface stiffness and shear lag leads to the shear strain localizations at the interfaces. The research results may provide useful information for the optimal design of CFRP laminates.

The strain measurement accuracy of the used Moiré method depends on the pitch accuracy of the fabricated specimen grid. From the straight and evenly spaced Moiré fringes and phases generated from the specimen grid at 0 MPa shown in Figure 7b–e, the fabricated grid is good without distortion and pitch variation, indicating the strain measurement is correct in this study. The grid fabricated by UV nanoimprint lithography has been proven to be suitable for deformation measurement [38]. The grid quality fabricated by UV nanoimprint lithography depends on the grid mold and the specimen surface flatness. Commercial grid molds and fine polishing process are suggested to be used for deformation measurement. Although the grid fabrication process by nanoimprint lithography is simple, the batch production technology of microgrids on specimens is still immature, which limits the wide application of the Moiré method. One of the future efforts is to improve the microgrid fabrication technique on various testing objects for accurate deformation measurement. Besides, periodic errors of the measured strain tend to occur in the developed Moiré method when the sampling pitch is not close to the specimen grid pitch, which is also a topic for future research.

Although the strain measurement accuracy, including the relative errors and the standard deviations of the developed Moiré method, has been verified from numerical simulation [33], the reproducibility of strain measurement of composite materials needs to be verified from batch experiments. To assure the reliability of the findings about the strain concentration and the influence of the specimen/grid manufacturing process on the strain distributions, statistical study from several specimens should be performed in the future work.

## 5. Conclusions

The interlaminar shear behavior of a [±45°]_4s_ laminated CFRP specimen in a three-point bending test was investigated from microscale strain distributions measured by a developed sampling Moiré technique. The full-field normal, shear and principal strain distributions of CFRP were measured. The tensile strain concentration regions pointed out several micro damage locations along the loading direction. The shear strain concentrations at interlaminar interfaces indicated the large shear deformations of different layers. The shear strain was positive at (45°/−45°) layer interfaces and negative at (−45°/45°) layer interfaces, indicating that the relative slip direction of 45° layers was right and of −45° layers was left, which was also visualized from the revised Moiré phases. The shear strain went up and then dropped with the load increase at the bottom (45°/−45°) layer interface, attributed to an emerged interlaminar damage. The shear strain distributions under different loads enable quantitative evaluation of the shear behavior and prediction of interlaminar damage, such as delamination of CFRP. In summary, despite this Moiré method requires a uniformly distributed grid before deformation, it can visually measure the specimen deformation with strong noise-resistance ability and is useful in detection and evaluation of tiny damages in composite materials.
